# Volumetric Absorptive Microsampling as an Alternative Tool for Biomonitoring of Multi-Mycotoxin Exposure in Resource-Limited Areas

**DOI:** 10.3390/toxins13050345

**Published:** 2021-05-11

**Authors:** Arnau Vidal, Lidia Belova, Christophe Stove, Marthe De Boevre, Sarah De Saeger

**Affiliations:** 1 Centre of Excellence in Mycotoxicology and Public Health, Department of Bioanalysis, Ghent University, Ottergemsesteenweg 460, B-9000 Ghent, Belgium; lidia.belova@uantwerpen.be (L.B.); Marthe.DeBoevre@UGent.be (M.D.B.); Sarah.desaeger@ugent.be (S.D.S.); 2 Laboratory of Toxicology, Department of Bioanalysis, Ghent University, Ottergemsesteenweg 460, B-9000 Ghent, Belgium; christophe.stove@ugent.be

**Keywords:** VAMS, multi-mycotoxin, mycotoxins, exposure, biomonitoring

## Abstract

Biomonitoring of biological samples arises as an effective tool to evaluate the exposure to mycotoxins in the population. Owing to the wide range of advantages, there is a growing interest in the use of non- and minimally invasive alternative sampling strategies, such as dried blood spot sampling or volumetric absorptive microsampling (VAMS). A VAMS-based multi-mycotoxin method was developed and validated for 24 different mycotoxins. Method validation was based on the Bioanalytical Method Validation Guideline of the Food and Drug Administration from the United States and for most of the studied mycotoxins, the results of the performance characteristics were in agreement with the criteria of the European Commission Decision 2002/657/EC. The recovery for the different mycotoxins was not haematocrit dependent and remained acceptable after storing the VAMS for 7 and 21 days at refrigeration temperature (4 °C) and room temperature, demonstrating that VAMS could be applied to assess mycotoxin exposure in blood in resource-limited areas, where there may be a delay between sampling and analysis. Finally, a comparison between VAMS and a procedure for liquid whole blood analysis, performed on 20 different blood samples, did not result in missed exposed cases for VAMS. Moreover, both methods detected similar levels of ochratoxin A, ochratoxin alpha, zearalenone and aflatoxin B1. Given all the benefits associated with VAMS and the developed method, VAMS sampling may serve as an alternative to conventional venous sampling to evaluate multiple mycotoxin exposure.

## 1. Introduction

Mycotoxins are present in a wide range of foods, from agricultural crops (maize, nuts, spices, wheat), cereal-based foods (baking products, pasta, breakfast cereals), beverages (fruits, juices and purees, beer and wine) and animal feed to dairy products [1,2,3]. Mycotoxin intake may lead to autoimmune illnesses, metabolic and biochemical deficiencies, allergic manifestations, reduction of reproductive efficiency, teratogenicity, carcinogenicity, mutagenicity, and even death [4]. However, mycotoxins as a group cannot be classified according to their toxicology or metabolism, as these vary depending on the different physicochemical properties and there is a wide range of mycotoxins with a great diversity in their modes of action [5].

Due to the large prevalence and toxicity of mycotoxins, biomonitoring of mycotoxin exposure arises as an effective tool to evaluate the risk of exposure among the population. Biomonitoring is most often performed by analysing mycotoxins or their metabolites in biological fluids such as urine and blood (whole blood, plasma, or serum) [6]. However, given the invasive nature associated with conventional blood sampling and the relatively large amounts of blood that are typically collected, this sampling procedure is not very appealing in practice. Besides, patients must go to a hospital or a doctor’s office for a venous blood draw. For this reason, there lies an increasing attraction in the use of non- and minimally invasive alternative sampling strategies for biomonitoring of contaminants’ exposure [7]. Examples include dried blood spots (DBS) sampling or volumetric absorptive microsampling (VAMS), which are associated with a wide range of advantages [8]. Besides allowing the patients to perform sampling themselves at home by a finger prick, these approaches can also be advantageous in countries where patients must move a long distance to clinical services. The small sample volume is another benefit, especially for specific populations, such as neonates and anaemic patients. In addition, the sampling procedure is accompanied by fewer difficulties with respect to sample handling, storage, and transport. All these advantages could be useful for biomonitoring mycotoxin exposure, especially in low and middle income countries, where mycotoxin exposure can be at dramatic levels [9] and equipment facilities are scarce [10].

A multi-mycotoxin DBS-based methodology was successfully validated by Osteresch et al. [11]. However, when compared with partial-punch DBS approaches, VAMS, in which a fixed volume of blood is absorbed by a polymeric absorbent tip, offers several advantages: the volume absorbed is not dependent on the haematocrit, there is no potential homogeneity issue [12,13,14] and, importantly, users indicated a preference towards VAMS [15]. Although VAMS has been applied for a multitude of analytes [16], including proteins such as haemoglobin [17], β-lactoglobulin and myoglobin [18], drugs [19] and contaminants such as perfluorinated compounds [20], to our knowledge, VAMS has never been tested for multiple mycotoxins analysis. 

Because of the many advantages associated with VAMS, this microsampling strategy might represent an important step forward for mycotoxin biomonitoring, especially in low- and middle income countries. Therefore, a VAMS-based multi-mycotoxin method was developed and validated for 24 mycotoxins: aflatoxin B1 (AFB1), aflatoxin B2 (AFB2), aflatoxin G1 (AFG1), aflatoxin G2 (AFG2), aflatoxin M1 (AFM1), α-zearalenone (α-ZEL), alternariol (AOH), alternariol monomethyl ether (AME), β-zearalenone (β-ZEL), deepoxy-deoxynivalenol (DOM-1), deoxynivalenol (DON), deoxynivalenol-3-glucoside (DON-3-glucoside), diacetoxyscirpenol (DAS), fumonisin B1 (FB1), fumonisin B2 (FB2), fumonisin B3 (FB3), HT-2 toxin (HT-2), nivalenol (NIV), ochratoxin A (OTA), ochratoxin alpha (OTα), roquefortin C (ROQ-C), T-2 toxin (T-2), T-2 triol toxin (T-2 triol) and zearalenone (ZEN). The method was validated following the guidelines of the European Commission’s Decision (EC) No. 2002/657 [21] and the Bioanalytical Method Validation described by the United States Department of Health and Human Services Food and Drug Administration [22]. The possible influence of hematocrit (Hct) was tested at 2 different mycotoxin concentrations by analysing all the mycotoxins at 3 different Hct levels. Moreover, VAMS devices were stored for 7 and 21 days refrigerated (4 °C) or at room temperature (RT, 20-23 °C) to evaluate the suitability of the method for remote areas where samples have to be stored for a long time before the analysis can be carried out. Finally, a small-scale clinical study was performed by analyzing 20 different blood samples and analyzing all samples using two different methodologies: via VAMS and analysis of the dried blood microsamples, or via conventional liquid/liquid extraction of whole blood. The described investigations aim at introducing VAMS as a valuable and additional tool to access multi-mycotoxin exposure in low- and middle-income countries. By validating the methodology for a high number and chemically diverse variety of mycotoxins, we provide an easily-applicable alternative to conventional invasive venous sampling of blood. To the authors’ knowledge, this is the first study validating the use of VAMS in the context of internal mycotoxin exposure assessment. The use of our proposed methodology is expected to further facilitate the identification of multiple mycotoxin exposure, particularly in remote areas or in special populations (e.g., children), and may help to elucidate associations between mycotoxin exposure and human adverse health outcomes.

## 2. Results

Recently, focus has been set towards human mycotoxicokinetics [23,24]. However, to date there is a lack of substantial investigation into the most optimal sampling period and sampling matrix to precisely assess mycotoxin exposure (i.e., urine, blood, faeces) [6,25]. Blood is envisaged to be the best matrix to assess exposure for some mycotoxins such as aflatoxins [26], however, blood sampling is an invasive technique, and the obtained samples are difficult to store without alterations. For this reason, based on an existing LC-MS/MS methodology to analyze 24 mycotoxins in blood [1,6,27], a microsample-based alternative was developed, by applying the VAMS technique. The mycotoxins included in this study were selected based on their toxicity, emerging profile and/or incidence in biomonitoring surveys. Noteworthy, other mycotoxin biomarkers which were not in our scope can be easily implemented, based on the wide physicochemical range of the mycotoxins investigated in this study.

### 2.1. Sample Preparation

As all VAMS references describe, a complete immersion of the device into the blood should be avoided, only the tip has to be dipped into the blood [18,28,29,30]. Different studies have not specified the time of the tip’s contact with the blood [24,31], or they used similar times as used in this study. However, it was preferred to establish a fixed time of 7 s to ensure full absorption to obtain a standardized protocol, as also used by [30,32,33,34]). After the tip was dried, it is generally recommended to wait ≥2–3 h to consider a tip as dry [34]. It is imperative for validation purposes to wait until the tip is dry as routine analysis will be done with dry tips. On the other hand, we do not advice to use high temperatures to dry as some mycotoxins could undergo degradation [35,36]. Moreover, the drying process should be in dark conditions as some mycotoxins are light-sensitive [37]. Although a relatively low volume (250 µL) of extraction solvent was used to extract the tips (containing 10.4 µL of blood), still, the dilution was too high to directly proceed to LC-MS/MS, necessitating the inclusion of an evaporation and reconstitution step to concentrate the sample. As the utilized extraction solvent (acetonitrile/water/acetic acid, 59/40/1, *v*/*v*/*v*) yielded good and consistent recoveries for the vast majority of the mycotoxins (see further), no other extraction solvent mixtures were tested. Among described VAMS studies, different extraction solvents have been used, typically being a mixture of an aqueous and an organic phase, at different proportions [28,38], depending on the analyte(s) of interest. The evaporation before LC-MS/MS analysis is a common step in mycotoxin studies from biological fluids as mycotoxin concentrations in urine or blood are usually very low. 

### 2.2. Optimization of the LC-MS/MS Conditions 

MS spectra were verified in both positive (ESI+) and negative ionization (ESI-) mode. A precursor ion for each analyte was selected and cone voltages were optimized. For all the mycotoxins the formation of the [M+MeOH+H]+, [M+H]+ or [M+NH4]+ adducts led to higher signal intensities, hence these adducts were chosen as precursor ions if they were obtained. The two most intense product ions were selected for the MRM transitions of the MS method after applying different collision energies (Table 1). Based on previous studies carried out in the same laboratory [24,39,40], an Acquity UPLC^®^ HSS T3 [1.8 µm, 2.1 mm × 100 mm] chromatographic column was chosen, demonstrating proper retention times for all the analyzed mycotoxins. Different mobile phases for an optimal separation, including mixtures of water with a volatile organic acid or ammonium acetate with an organic solvent (methanol) were tested [24,41]. The results showed that an optimal separation with the best sensitivity was obtained with water/methanol/acetic acid (94/5/1, *v*/*v*/*v* (A)) and methanol/water/acetic acid (97/2/1, *v*/*v*/*v* (B)), both adjusted with 5 mM ammonium acetate. These results are in accordance with previously reported LC-MS/MS methods for multi-mycotoxin analysis [40].

### 2.3. Method Validation

The multi-mycotoxin VAMS-based LC-MS/MS methodology was validated following the criteria mentioned in Commission Decision (EC) No. 2002/657 [21] and based on the Bioanalytical Method Guidance for Industry elaborated by the FDA [22].

### 2.4. Specificity and Calibration Curve 

There were no detectable peaks or possible interferences for the identification and quantification of the target compounds in the ± 2.5% margin of the relative retention time. Hence, the developed method can be considered specific. Concerning the calibration curves, unweighted linear calibration models were accepted for all analyzed mycotoxins because all back-calculated concentrations lay within 15% of the corresponding nominal concentrations. A plot was constructed of the residuals versus concentration, and the error was randomly distributed around the concentration axis for all mycotoxins, indicating the absence of proportional and systematic errors.

### 2.5. Limit of Detection and Limit of Quantification

The LOD and LLOQ obtained for all the studied mycotoxins in the developed method are shown in Table 2. LOD and LLOQ are critical parameters within the analysis of mycotoxins in biological fluids. Although, owing to the small volume (10.4 µL), the LODs for the VAMS-based method are somewhat higher than those obtained via liquid-liquid extraction of larger volumes of blood [42,43], these are still close to the LODs of other multi-mycotoxin studies that use liquid-liquid extraction. For instance, starting from 100 µL blood, a similar LOD was obtained for AFB1 (0.04 ng/mL), 20% lower LOD for AFB2 (20 ng/mL) and 30% lower LOD for AFG1 and AFG2 (0.07 ng/mL) [44]. The LOD we obtained for NIV was 50% lower. Degen et al., (2018) [23] obtained 50% and 25% lower LODs for OTα and OTA, respectively, compared with the values presented in this paper. So, the acquired LOD and LLOQ levels are similar to those obtained with other multi-mycotoxin methods and permit to perform multi-mycotoxin analysis in blood. However, further research with VAMS could allow a further lowering of the LODs, to achieve even better results.

### 2.6. Apparent Recovery, Intraday Precision (Repeatability), Interday Precision (Reproducibility) and Measurement Uncertainty

**Figure 1:**
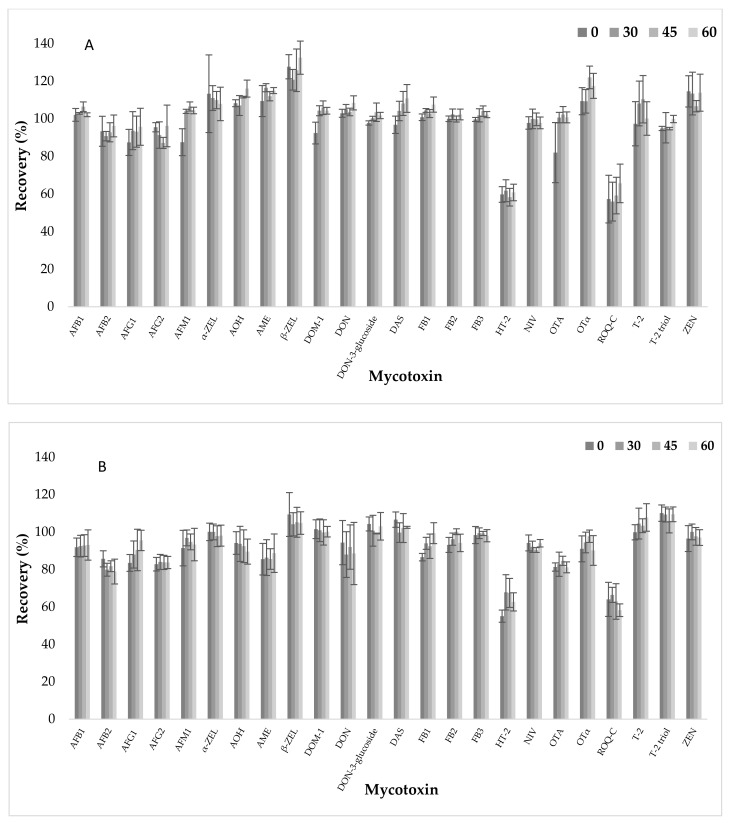
Mycotoxin recovery (%) and standard deviation (bars) using VAMS for 3 different haematocrit levels (30, 45 and 60) at (**A**) low and (**B**) high mycotoxin concentration. Mycotoxin concentrations at low concentration: 0.5 ng/mL (aflatoxin B1 (AFB1), B2 (AFB2), G1 (AFG1), M1 (AFM1), G2 (AFG2), ochratoxin A (OTA) and ochratoxin alpha (OTα)), 2.5 ng/mL (deepoxy-deoxynivalenol (DOM-1), deoxynivalenol (DON), deoxynivalenol-3-glucoside (DON-3-glucoside), diacetoxyscirpenol (DAS), HT-2 toxin (HT-2) and T-2 toxin (T-2)) and 10 ng/mL (alpha-zearalenone (α-ZEL), alternariol (AOH), alternariol monomethyl ether (AME), beta-zearalenone (β-ZEL), fumonisin B1 (FB1), B2 (FB2), B3 (FB3), nivalenol (NIV), roquefortin C (ROQ-C), T-2 triol toxin (T-2 triol) and zearalenone (ZEN)). Mycotoxin concentrations at high concentration: 12.5 ng/mL (aflatoxin B1 (AFB1), B2 (AFB2), G1 (AFG1), M1 (AFM1), G2 (AFG2), ochratoxin A (OTA) and ochratoxin alpha (OTα)), 62.5 ng/mL (deepoxy-deoxynivalenol (DOM-1), deoxynivalenol (DON), deoxynivalenol-3-glucoside (DON-3-glucoside), diacetoxyscirpenol (DAS), HT-2 toxin (HT-2) and T-2 toxin (T-2)) and 250 ng/mL (alpha-zearalenone (α-ZEL), alternariol (AOH), alternariol monomethyl ether (AME), beta-zearalenone (β-ZEL), fumonisin B1 (FB1), B2 (FB2), B3 (FB3), nivalenol (NIV), roquefortin C (ROQ-C), T-2 triol toxin (T-2 triol) and zearalenone (ZEN)).

Apparent recovery, intra and interday precision and measurement uncertainty were calculated for the 24 studied mycotoxins (Figure 1 and Table 2). Most of the studied mycotoxins were effectively recovered. Only β-ZEL had an average apparent recovery far above 100%. β-ZEL is an important phase I metabolite of ZEN produced during in vitro microsomal studies [45]. α-ZEL is the most predominant metabolite produced in vitro in phase I metabolism and it is 92-fold more estrogenic than ZEN, while β-ZEL has a 2.5 times lower potency [46,47]. Relatively low average apparent recoveries were observed for HT-2 and ROQ-C, at 46 and 56%, respectively. The low recovery of HT-2 was not caused by a higher T-2 recovery. Although ideally a higher recovery would be obtained for HT-2, as HT-2 toxin is one of the main T-2 metabolites in animals and humans [6], the LOD we obtained was still low enough as to be detected in human blood samples (0.74 ng/mL). ROQ-C on the other hand, is barely present in food [48]. In initial experiments, acetyl-deoxynivalenols (3-acetyldeoxynivalenol and 15-acetyldeoxynivalenol) were also included in the analyte panel. Owing to the well-known transformation of acetyl-deoxynivalenols to DON [49], this resulted in recoveries of DON up to 200%, while acetyl-deoxynivalenol recovery values were reduced to around 50%. As acetyl-deoxynivalenol forms are rapidly transformed to DON during digestion [50], these compounds are not -or only minimally- expected in blood and were hence not included in this validation. This allowed to achieve good validation and uncertainty measurement results for DON. The measurement uncertainty was in some cases considerably high, for example for β-ZEL it was 79% for the lowest assayed concentration. It is difficult to compare the measurement uncertainty obtained in this study with other multi-mycotoxin methods in plasma or blood because this parameter is generally not presented in these studies. However, this large uncertainty suggests that, in the context of multi-mycotoxin analysis, for some compounds the use of VAMS should be considered as a screening methodology rather than as a tool to obtain definitive quantitative results.

### 2.7. Matrix Effect

A considerable matrix effect (SSE; signal suppression or enhancement) was detected for most of the analyzed compounds, with values ranging between 8.61 and 124% (Table 2). However, in almost all instances the applied IS could adequately compensate for the differences in ionization (Table 2) [51]. SSE is commonly found during mycotoxin analysis in blood or plasma and similar matrix effects have been observed in other studies. Lauwers et al., (2019) [52] analyzed 24 mycotoxins with DBS and found a SSE range between 60 and 112%, Osteresch et al., (2017) [11] reported values ranging from 14% to 939% upon analysis of 26 mycotoxins with DBS, and Slobodchikova et al., (2018) [44] reported a SSE range between 35 and 110%, analyzing 17 mycotoxins in plasma using protein precipitation as analysis method. Although internal standards (IS) may adequately compensate for SSE, a point of attention -certainly when dealing with suppression of ionization- will be that adequate sensitivity is maintained; monitoring of the absolute signal heights of the IS can be used for this purpose. 

### 2.8. Stability 

Short- and long-term stabilities were assessed by analyzing low and high concentrations in duplicate after storage for 7 and 21 days at different temperatures (Table 3). The recoveries obtained after 7 and 21 days were satisfactory (between 70‒120%) for most of the analyzed mycotoxins. The good stability results imply that VAMS can be used to assess mycotoxin exposure in blood in resource-limited areas, where samples may have to be collected under remote conditions, where a substantial time delay exists between sampling and analysis. These findings are in line with mycotoxin stability studies in DBS samples [11], where a good stability was observed at fridge or freezing temperature for 24 weeks, but recoveries started to decline (>30%) after 5 weeks of storage at room temperature. In most instances, however, it should be possible to perform the analysis or to store samples at lower temperature within 3 weeks. Hence, our stability studies, in which samples were stored for up to 21 days, show that VAMS is a highly promising sampling methodology. The introduction of VAMS or DBS can lead to a shift of matrix of analysis (to blood over urine). Increasing the use of blood analysis for mycotoxin exposure studies could be an important step forward because urine analysis, though representing a reliable tool to determine the exposure of mycotoxins as DON [24], is not the correct option for many other mycotoxins. For example, only 1% of FBs and 26% of ZEN are excreted via urine, respectively [53]. Also, the determination of AFB1-lysine in blood is considered the most reliable biomarker for chronic aflatoxin exposure [54].

### 2.9. Hematocrit Assessment

Hct is discussed as a key factor influencing the quantitative determination of compounds in DBS [55] and any DBS-based method should evaluate Hct as a variable, as Hct will influence the spreading of blood on filter paper (and hence the amount of blood contained in a partial punch), and may influence recovery and/or matrix effects as well [56,57]. By absorbing a fixed blood volume irrespective of the Hct, VAMS overcomes the Hct issue associated with partial-punch DBS analysis, as VAMS does not suffer from issues related to differential spreading or inhomogeneity [13,14]. However, VAMS may still suffer from extractability issues linked to the Hct [13,16,57], and, hence, it is essential that a possible impact of Hct on the recovery is evaluated, as was done for e.g., caffeine and paraxanthine [13], paracetamol [38], cobalt [58] and anti-epileptic drugs [28], among others. We evaluated a possible impact of Hct at 3 different Hct levels at 2 different concentrations. As shown in Figure 1, Hct did not affect the recovery for any of the tested mycotoxins at the 3 different Hct levels (0.3, 0.4 and 0.6), supporting the robustness of the extraction. The present study is the first to quantify mycotoxins using VAMS, however, DBS have already been used to analyze mycotoxins [11,59,60,61]. DBS analysis of mycotoxins showed some Hct-dependence. While OTA concentrations were not impacted by the Hct [60], lower concentrations (p < 0.05) were found for β-ZAL, AFB1 and AFM1 at the lowest tested Hct (0.26), in a panel of 24 mycotoxins [59]. 

### 2.10. Application to Real Samples

Five mycotoxins and metabolites (OTA, OTα, ZEN, α-ZEL and AFB1) were detected in 20 analyzed blood samples (Table 4), yielding similar results for the VAMS-based and the liquid whole blood-based methodologies. It should be noted, though, that the lower LLOQ for the liquid whole blood-based procedure resulted in three ‘false negatives’ for OTA with the VAMS-based procedure (60% vs. 75% positives). The high degree of detection of OTA in blood samples was expected, given the known high exposure to OTA among the European population [42,62] and given its long elimination half-life in humans (35 days) [63]. Importantly, the VAMS-based procedure was equally effective in detecting the OTA metabolite, OTα, which is formed by cleavage of the phenylalanine moiety of OTA and is considered as one of the predominant metabolites of OTA in animals [6]. Several human plasma analyses indeed showed that OTα can have higher concentrations than OTA [64]. Hence, inclusion of this analyte ensured that no OTA-exposed cases were missed via the VAMS-based procedure. Relevant to mention is that similar levels of OTα were observed, irrespective of whether dried (VAMS) or liquid blood was analyzed (0.83 ± 0.21 vs. 0.78 ± 0.29 ng/mL). ZEN was detected in the same samples using both extraction methods and, moreover, at similar concentration levels: 8.05 ± 5.02 ng/mL in VAMS samples and 7.68 ± 4.81 ng/mL in liquid whole blood. ZEN is a common mycotoxin in cereal products and some studies pointed out that parts of the European population could exceed the tolerable daily intake (TDI): in Portugal 24% of studied population exceeded the TDI [65]. α-ZEL is one of the most important ZEN metabolites and in vitro studies with rats, pigs, goats, cows and humans showed that the yield of α-ZEL is much higher than that of β-ZEL [45]. This ZEN metabolite was detected in only one liquid blood sample, at a concentration below the LLOQ of the VAMS-based procedure. Also here, as ZEN had already been detected using the VAMS-based procedure, no ZEN-exposed case was missed when using VAMS. Finally, AFB1 was detected in 2 samples (Table 4) with similar values obtained for both methods. AFB1 is considered the most toxic mycotoxin and is classified as a group 1 carcinogenic agent (carcinogenic to humans) by the International Agency for Research on Cancer (IARC) [66]. In conclusion, despite the fact that the VAMS-based procedure had a somewhat higher LOD and LLOQ for some mycotoxins than the liquid whole blood-based procedure, no exposed cases were missed in this cohort, when applying VAMS. Although these findings should be corroborated by a follow-up study, using larger cohorts, these findings suggest that blood collection with VAMS could be feasible to reliably assess mycotoxin exposure. This is important as exposure assessment of some mycotoxins -such as OTA- can be better estimated through blood analysis [6]. While other microsampling techniques have been applied for multi-mycotoxin analysis, such as DBS [11,52], the use of VAMS is preferred because a fixed volume of blood is absorbed, the volume absorbed is not dependent on the haematocrit, there is not a potential homogeneity issue [12,13,14] and, importantly, users indicated a preference towards VAMS [15].

## 3. Conclusions

To the best of our knowledge, this is the first application of VAMS for multiple mycotoxin analysis. Although the uncertainty measurement was high for some mycotoxins, the developed method could be considered successfully validated for the most commonly present mycotoxins as DON, OTA, AF and FB. Moreover, VAMS could offer some advantages compared to other alternative blood microsampling techniques such as DBS sampling. Absorption of blood via VAMS is Hct-independent, and, importantly, as demonstrated here, also the extraction procedure we used was Hct-independent. Finally, in a cohort of 20 authentic samples, similar results were achieved when comparing a VAMS-based and a liquid whole blood-based procedure, with no exposed cases being missed by the VAMS-based procedure. Given all the benefits offered by VAMS and the robust method that was developed, VAMS sampling can serve as an excellent alternative to conventional venous sampling to perform a quantitative screening of mycotoxin exposure. Our findings provide a solid basis for future studies, using larger patient cohorts, with sampling via direct fingerpick. In addition, this microsampling approach will further help to elucidate the impact of chronic exposure to multiple mycotoxins on human health, allowing associations to be made with adverse health outcomes, taking into account the toxicokinetic profiles of the observed mycotoxins. 

## 4. Materials and Methods

### 4.1. Chemicals and Reagents

The individual mycotoxin solid calibration standards (1 mg) of DON, DON-3-glucoside, 3-acetyldeoxynivalenol (3-ADON), 15-acetyldeoxynivalenol (15-ADON), DOM-1, ZEN, α-ZEL, β-ZEL, T-2, T-2 triol, HT-2, DAS, AOH, AME, NIV, OTA, OTα, AFB1, AFB2, AFG1, AFG2, AFM1, FB1, FB2, FB3 and ROQ-C and internal standards (isotope-labelled DON, ZEN, AFB1 and FB1) were obtained from Sigma-Aldrich (Bornem, Belgium). All mycotoxin solid standards were dissolved in methanol (1 mg/mL) and were storable for a minimum of 1 year at −18 °C [48]. The mycotoxin working solutions and IS were prepared in methanol, and stored at −18 °C. Water was obtained from an Aurim^®^ Pro water system from Sartorius (Brussels, Belgium). Disinfectol^®^ (denaturated ethanol with 5% ether) was supplied by Chem-Lab (Zedelgem, Belgium). Methanol (LC-MS grade) was purchased from BioSolve (Valkenswaard, The Netherlands), while acetonitrile (Analar Normapur) and ammonium acetate were obtained from VWR International (Zaventem, Belgium). Acetic acid (glacial, 100%) and formic acid (98‒100%) were supplied by Merck (Darmstadt, Germany). VAMS devices (Mitra^TM^) were obtained from Neoteryx (Torrance, CA, USA).

### 4.2. Sample Collection, Sample Preparation and Extraction Procedure

EDTA-anticoagulated blood samples for method development and validation purposes were supplied by Rode Kruis Vlaanderen (Ghent, Belgium). These were kept at −80 °C until use. One mL of a blood sample was poured in a glass tube by fortifying (spiking) at five different levels depending on the mycotoxin (Table 1). The samples were left for a 10 min equilibration at RT. Then, samples were prepared by dipping the tip into spiked whole blood. Overfilling of the devices was prevented by not completely immersing the tip into the blood. After the tip was completely coloured, the contact with the blood surface was extended for 7 s to ensure full absorption, as described previously [28]. After completely filling the tips, the devices were dried in the accompanying clamshells for ≥3 h at room temperature until ultra-performance liquid chromatography - tandem mass spectrometry (UPLC^®^-MS/MS) analysis. Sample preparation was performed by separating the VAMS tips from the plastic handlers and transferring these into 2 mL Eppendorf tubes. Subsequently, the extraction was carried out by adding 250 µL extraction solvent (acetonitrile/water/acetic acid, 59/40/1, *v*/*v*/*v*) containing the internal standards at a concentration of 0.25 ng/mL ^13^C_17_ –AFB1 and 25 ng/mL for ^13^C_15_ –DON, FB1 and ZEN. Afterwards, ultra-sonication was undertaken for 20 min and samples were shaken for 30 min at room temperature using an overhead shaker (Agilitec, J. Toulemonde and Cie, Paris, France). The tips were removed, and the supernatant was evaporated to dryness under a gentle stream of nitrogen using a Turbovap LV Evaporator (Biotage, Charlotte, USA). Extracts were reconstituted in 50 µL of injection solvent (methanol/water, 60/40, *v*/*v*), vigorously vortexed and subjected to centrifugation (Ultrafree^®^-MC centrifugal device, Millipore, Bedford, MA, USA) for 10 min at 5000 g. Finally, samples were transferred into vials for analysis and 5 µL were injected into the UPLC^®^-MS/MS-system. 

### 4.3. UPLC-MS/MS Analysis

A Waters Acquity UPLC^®^ system coupled to a Quattro XEVO TQS mass spectrometer (Waters, Manchester, UK) was used to analyze the blood samples. Data acquisition and processing was performed with MassLynx™ version 4.1 and QuanLynx^®^ version 4.1 software (Waters, Manchester, UK). A Waters Acquity UPLC^®^ HSS T3 (2.1 × 100 mm, 1.8 µm) column was applied (Waters, Manchester, UK). Two different mobile phases were used and consisted of water/methanol/acetic acid (94/5/1, *v*/*v*/*v* (A)) and methanol/water/acetic acid (97/2/1, *v*/*v*/*v* (B)), both adjusted with 5 mM ammonium acetate. The gradient elution program started at 5% mobile phase B, which was increased linearly to 65% in 7 min. Then, in 4 min, mobile phase B increased to 75%, after which it further increased to 99%, which was maintained for 1 min before reintroducing a 4-min equilibration step, resulting in a total run time of 16 min. The flow rate was set at 0.3 mL/min. The MS was operated in both positive and negative electrospray ionisation mode (ESI+/ ESI−). The capillary voltage was 30 kV, and nitrogen was applied as nebulizer gas. The source and desolvation temperatures were set at 150 °C and 200 °C, respectively. The argon collision gas pressure was 9 × 10^−6^ bar, the cone gas flow 150 L/h and the desolvation gas flow 550 L/h. Two selected reaction monitoring (SRM) transitions with a specific dwell-time were optimised for each analyte, in order to increase the sensitivity and the selectivity of the MS conditions (Table 2).

### 4.4. Method Validation

The method was validated to meet the criteria of the European Commission Decision 2002/657/EC [21] and was based on the Food and Drug Administration guidelines for bioanalytical method validation [22]. Blood samples, considered blank, were spiked and used for the validation. The following set of parameters was used to examine the method performance: specificity, calibration curve, apparent recovery, intraday (RSDr) and interday precision (RSDR), measurement uncertainty (U), limit of detection (LOD), lower limit of quantification (LLOQ), matrix effect and stability. According to Commission Decision (EC) No 2002/657, laying down the performance criteria of analytical methods, four identification points should be satisfied to achieve confirmation of the identity of the detected compound: 1 precursor and at least 2 product ions should be controlled, the relative intensities of the detected ions should rest within accepted deviations to those of the calibration, detected ions should have a signal-to-noise ratio (S/N) of at least 3 and the relative retention time of the detected ions must rest within a margin of 2.5%.

### 4.5. Specificity

Specificity was evaluated by analysis of five blank VAMS samples on five different days (*n* = 25). The chromatograms were assessed to recognise probable interferences for the identification and quantification of the selected mycotoxins. Selectivity/specificity was considered acceptable if there were no interfering peaks in the 2.5% margin of the relative retention time. 

### 4.6. Calibration Curves

Calibration curves in neat solvent were made for all validation runs (*n* = 5). These curves were used for the evaluation of the best fitting calibration model. The model with the lowest sum % residual error was selected. To accept this model, back-calculation of the calibrators should yield results within ±15% of the nominal concentration. Homoscedasticity and the calibration model were evaluated by generating five 5-point calibration curves. Homoscedasticity was tested by performing an F-test (α = 1%) at the different concentrations points. Furthermore, for the calibration model, unweighted linear and quadratic regression were performed to find the best fitting model. 

### 4.7. Limit of Detection and Lower Limit of Quantification

LOD and LLOQ (ng/mL) were determined according to the guidelines of the International Conference of Harmonisation (ICH, 2005) and were based on the standard deviation of the y-intercept and the slope. Therefore, blank blood samples were spiked in decreasing concentrations within the range based on expected LOD and LLOQ levels, determined during method optimization. The experiment was conducted in three independent replicates. The standard deviation of the y-intercept as well as the slope of the curve were calculated using a linear estimation through the method of least squares. LOD equals 3.3 times the residual standard deviation of the regression line (standard error of the predicted response for each concentration in the regression) divided by the slope. LLOQ equals 6 times the residual standard deviation of the regression line divided by the slope. 

### 4.8. Apparent Recovery

Blank blood samples were spiked at five different concentration levels (Table 1) and used to generate VAMS samples, which were analysed in triplicate on five different days. *Apparent recovery* (Rapp, %) was achieved by contrasting the acquired concentrations (calculated with the aid of the analysed calibration curve in neat solvent) to the spiked concentrations. Then, the mean concentration and the standard deviation were calculated out of the analysis of the spiked samples per concentration level

(1) $$Apparent\;recovery\;\left(\%\;\right)\;=\;\left(\frac{\mathrm{Mean}\;\mathrm{observed}\;\mathrm{concentration}\;\left(\frac{\mathrm{ng}}{\mathrm{mL}}\right)}{\mathrm{Spiked}\;\mathrm{concentration}\;\left(\frac{\mathrm{ng}}{\mathrm{mL}}\right)}\right)\;*\;100$$

### 4.9. Intraday Precision (Repeatability (RSDr)) and Interday Precision Intra-Laboratory (Reproducibility (RSDR))

Blank blood samples were spiked at five different concentration levels and used to generate VAMS samples that were analysed in triplicate on five different days. The mean and standard deviation of the obtained concentrations (calculated with the aid of the analysed calibration curve in neat solvent) were calculated per concentration level. The repeatability and reproducibility of the method were expressed as the variation coefficient (*VC* %) (Equation (2)). The criterion to consider the RSDr and RSDR acceptable was <20%.

(2) $$VC\;\left(\%\right)\;=\;\left(\frac{\mathrm{Standard}\;\mathrm{deviation}\left(\frac{\mathrm{ng}}{\mathrm{mL}}\right)}{\mathrm{mean}\;\mathrm{observed}\;\mathrm{concentration}\left(\frac{\mathrm{ng}}{\mathrm{mL}}\right)}\right)\;*\;100$$

### 4.10. Measurement Uncertainty (U)

The measurement uncertainty of the protocol was quantified applying the RSD*_R_* and the bias of the method (Equation 3). The combined standard uncertainty (*u_c_*) equals the positive square root of the interday precision and the bias of the analytical method, which contains the uncertainty of the purity of the used standards (*U*_[*Cref*]_), the accuracy of the bias (*S_bias_*) and the root mean square of the bias (*RMS_bias_*). The combined expanded measurement uncertainty, manifested as *U*, was achieved by multiplying the standard measurement uncertainty by a coverage factor (k = 2) and provides a range that involves the result with 95% confidence

(3) $$U\;\left(\%\right)\;=\;2\;*\;u_{c}=\surd \left[{\left({\mathrm{RSD}}_{R}\right)}^{2}+U_{\left[Cref\right]}^{2}+S_{bias}^{2}+RMS_{bias}^{2}\right]$$

### 4.11. Matrix Effect

Matrix effects are caused by a competition process, occurring during ionization, between the analyte ions and co-eluting matrix compounds, which are not detected but also form ions in the LC-MS/MS interface. The ionization of the compound of interest can either be improved or suppressed (signal suppression or enhancement, *SSE*), depending on the ionization efficiency of the analytes and the (co-eluting) matrix compounds. The matrix effect was calculated as the ratio of the calibration curve slope in post-extraction spiked blank blood VAMS extract against the calibration curve slope in neat solvent, processed without (Equation (4)) and with IS compensation (Equation (5)).

(4) $$SSE\;=\;\left(\frac{\mathrm{Slope}\;\mathrm{spiked}\;\mathrm{extract}}{\mathrm{Slope}\;\mathrm{standard}}\right)$$

(5) $$SSE\;=\;\left(\frac{\mathrm{Slope}\;\mathrm{spiked}\;\mathrm{extract}\;\mathrm{compensated}\;\mathrm{by}\;\mathrm{IS}}{\mathrm{Slope}\;\mathrm{standard}\;\mathrm{compensated}\;\mathrm{by}\;\mathrm{IS}}\right)$$

### 4.12. Stability Trial

The stability of mycotoxins in VAMS samples was tested for different durations of storage (7 and 21 days) and temperatures (room temperature and refrigeration, 4 °C), in the dark, at 3 different concentrations, evaluated in duplicate (*n* = 6) for each condition (Table 3). Based on these values, the mean degradation ratio was calculated by comparing the results from the different assayed conditions to recovery samples obtained in the validation study (‘fresh samples’). 

### 4.13. Hematocrit Level

The impact of the Hct on the recovery was evaluated following the instructions described by [55]. Therefore, 2 different concentrations at 3 different Hct levels (0.30, 0.45 and 0.60) were tested. The 3 different Hct levels were prepared by centrifuging an aliquot of blood with a Hct of 0.51 in Eppendorf tubes for 6 min at 2000 g and by removing or adding plasma. All samples were analyzed against a calibration curve with VAMS samples derived from spiked blood with Hct 0.45.

### 4.14. Clinical Study

In order to objectively indicate the validity of the obtained results, 20 different EDTA-anticoagulated blood samples (5 mL) were collected from Rode Kruis (Ghent, Belgium). The use of the human samples was approved by the Ethics Committee of Ghent University Hospital (B670201630414). Each sample was analyzed following 2 different protocols: (1) the VAMS methodology as described in this manuscript and (2) a liquid/liquid multi-mycotoxin extraction methodology, applied on liquid whole blood [44]. In brief, to 100 µL of blood, previously spiked with IS, 100 µL of acetonitrile was added in a 2 mL Eppendorf tube. Then, samples were vortexed for 20 s and shaken using an overhead shaker (Agitelec, J. Toulemonde and Cie, Paris, France) for 30 min. The Eppendorfs were then centrifuged at 9000× *g* for 6 min. Next, 160 µL of the supernatant was evaporated to dryness (N2, 40 °C). Finally, the dry residue was redissolved in 100 µL of the injection solvent (methanol/water, 60/40, *v*/*v*), vigorously vortexed and subjected to centrifugation (Ultrafree^®^-MC centrifugal device, Millipore, Bedford, MA, USA) for 10 min at 5000 g. Finally, samples were transferred into vials for analysis and 5 µL were injected into the LC-MS/MS.

### 4.15. Statistical Analysis

Statistical analysis was carried out operating with Microsoft Office Excel 2007 (Redmond, WA, USA) and SPSS^®^ 15.0 (Chicago, IL, USA). The paired t-test (*r* < 0.05) was applied to explore probable mycotoxin concentration variances in the stability trial.

## Figures and Tables

**Table 1:** Optimized LC-ESI-MS/MS parameters for the confirmation and quantification of: aflatoxin B1, aflatoxin B2, aflatoxin G1, aflatoxin G2, aflatoxin M1, alpha zearalenone, alternariol, alternariol methyl ether, beta zearalenone, deepoxy-deoxynivalenol, deoxynivalenol, deoxynivalenol-3-glucoside, diacetoxyscirpenol, fumonisin B1, fumonisin B2, fumonisin B3, HT-2 toxin, nivalenol, ochratoxin A, ochratoxin alpha, roquefortin C, T-2 toxin, T-2 triol toxin, zearalenone, and the isotope-labelled internal standards (^13^C_17_) aflatoxin B1, (^13^C_15_) deoxynivalenol, (^13^C_34_) fumonisin B1 and (^13^C_18_) zearalenone.

Mycotoxin	Precursor Ion (*m/z*)	Product Ions (*m/z*) Quantifier/Qualifier	CE (eV)	CV (v)	Retention Time (min)
AFB1	313.0	241.1/285.0	32/20	65	6.93
AFB2	315.0	259.0/287.0	28/24	25	6.65
AFG1	329.0	243.0/311.0	24/20	50	6.28
AFG2	331.0	285.0/313.0	28/24	40	5.97
AFM1	329.1	259.1/273.1	25/22	30	6.02
α-ZEL	321.1	175.1/177.0	22/17	30	9.29
AOH	258.9	185.1/213.1	30/26	40	8.12
AME	272.9	199.3/258.2	30/29	57	10.25
β-ZEL	321.5	177.3/189.1	15/20	30	8.50
DOM-1	281.1	215.1/233.1	9/9	40	4.43
DON	297.0	231.0/249.0	9/9	40	3.21
DON-3-glucoside	476.1	249.0/297.0	16/12	15	3.08
DAS	384.1	247.1/307.1	12/9	35	7.11
FB1	722.1	334.2/352.1	36/32	40	8.28
FB2	706.1	336.2/354.2	36/30	70	10.59
FB3	706.2	354.3/530.2	30/28	70	9.67
HT-2	447.0	285.0/345.0	20/18	40	7.98
NIV	313.0	175.0/177.0	21/16	30	2.55
OTA	403.9	358.0/238.9	12/20	40	9.33
OTα	257.0	221.1/239.1	20/10	30	5.83
ROQ-C	390.1	193.0/322.0	24/24	25	8.35
T-2	484.3	215.2/305.2	18/12	40	8.82
T-2 triol	405.2	125.2/303.1	14/14	30	7.17
ZEN	319.2	283.1/301.1	12/10	40	9.64
^13^C_17_ –AFB1	330.0	285.0/301.0	26/22	40	6.94
^13^C_15_ –DON	311.9	103.4/245.2	10/10	30	3.21
^13^C_34_ –FB1	756.4	356.2/374.2	40/36	40	8.28
^13^C_18_ –ZEN	337.3	199.1/214.9	22/22	40	9.65

CE: Collision energy. CV: Cone Voltage. Aflatoxin B1 (AFB1), aflatoxin B2 (AFB2), aflatoxin G1 (AFG1), aflatoxin G2 (AFG2), aflatoxin M1 (AFM1), α-zearlenone (α-ZEL), alternariol (AOH), alternariol monomethyl ether (AME), β-zearalenone (β-ZEL), deepoxydeoxynivalenol (DOM-1), deoxynivalenol (DON), deoxynivalenol-3-glucoside (DON-3-glucoside), diacetoxyscirpenol (DAS), HT-2 toxin (HT-2), fumonisin B1 (FB1), fumonisin B2 (FB2), fumonisin B3 (FB3), nivalenol (NIV), ochratoxin A (OTA), ochratoxin α (OTα), roquefortin C (ROQ-C), T-2 toxin (T-2), HT-2 toxin (HT-2), T-2 triol toxin, zearalenone (ZEN), and isotope-labelled internal standards (^13^C_17_) aflatoxin B1 (^13^C_17_ –AFB1), (^13^C_15_) deoxynivalenol (^13^C_15_ –DON), (^13^C_34_) fumonisin B1 (^13^C_34_ –FB1) and (^13^C_18_) zearalenone (^13^C_18_ –ZEN).

**Table 2:** Results for the limit of detection (LOD), lower limit of quantification (LLOQ), matrix effect (%), apparent recovery (Rapp) and measurement uncertainty (U) for all the analysed mycotoxins (aflatoxin B1, aflatoxin B2, aflatoxin G1, aflatoxin G2, aflatoxin M1, alpha zearalenone, alternariol, alternariol methyl ether, beta zearalenone, deepoxy-deoxynivalenol, deoxynivalenol, deoxynivalenol-3-glucoside, diacetoxyscirpenol, fumonisin B1, fumonisin B2, fumonisin B3, HT-2 toxin, nivalenol, ochratoxin A, ochratoxin alpha, roquefortin C, T-2 toxin, T-2 triol toxin and zearalenone).

Mycotoxin	LOD (ng/mL)	LLOQ (ng/mL)	Absolute Matrix Effect (%)		Concentration (ng/mL)	Rapp (%)	U (%)
			Analyte	IS Compensated			
AFB1	0.04	0.07	15.3	102	0.50	104	24.0
					1.25	96.1	22.1
					2.50	84.6	32.4
					5.00	91.3	35.6
					12.5	88.9	39.9
AFB2	0.13	0.26	15.1	97.9	0.50	76.9	41.9
					1.25	88.4	22.8
					2.50	90.0	34.2
					5.00	95.8	28.4
					12.5	76.3	37.8
AFG1	0.12	0.24	13.8	101	0.50	70.1	12.1
					1.25	89.8	18.2
					2.50	86.3	27.9
					5.00	92.1	19.4
					12.5	82.8	24.6
AFG2	0.15	0.30	12.9	92.1	0.50	81.0	26.3
					1.25	95.1	25.6
					2.50	87.2	19.5
					5.00	94.3	12.3
					12.5	79.9	17.6
AFM1	0.13	0.26	15.2	100	0.50	113	13.9
					1.25	110	19.8
					2.50	100	25.3
					5.00	109	17.9
					12.5	92.5	19.3
α-ZEL	2.64	5.30	15.5	107	10.0	148	35.3
					25.0	110	24.1
					50.0	114	13.3
					100	111	12.7
					250	106	14.1
AOH	1.37	2.74	13.2	105	10.0	136	49.1
					25.0	124	43.1
					50.0	138	42.8
					100	127	48.2
					250	92.8	30.1
AME	1.86	3.72	10.4	83.1	10.0	124	57.2
					25.0	124	42.1
					50.0	134	40.7
					100	118	42.1
					250	89.3	32.7
β-ZEL	6.76	13.52	14.0	106	10.0	181	79.2
					25.0	122	73.9
					50.0	149	40.4
					100	151	45.7
					250	115	33.5
DOM-1	0.57	1.14	42.5	94.5	2.50	100	14.9
					6.25	101	12.9
					12.5	110	16.5
					25.0	113	14.6
					62.5	101	15.2
DON	0.39	0.78	47.9	96.2	2.50	113	15.9
					6.25	93.4	15.6
					12.5	106	18.0
					25.0	105	14.1
					62.5	98.9	18.5
DON-3-glucoside	0.85	1.70	50.2	97.4	2.50	126	54.6
					6.25	118	18.5
					12.5	113	19.9
					25.0	115	18.0
					62.5	102	15.6
DAS	0.85	1.71	16.6	109	2.50	109	29.3
					6.25	103	21.3
					12.5	104	20.6
					25.0	113	18.0
					62.5	101	17.7
FB1	1.54	3.09	10.0	103	10.0	111	19.8
					25.0	94.3	16.9
					50.0	87.5	12.5
					100	89.6	13.5
					250	88.7	13.1
FB2	0.97	1.94	78.1	106	10.0	91.6	37.3
					25.0	90.7	31.0
					50.0	116	36.1
					100	95.8	17.9
					250	93.1	19.5
FB3	1.06	2.12	8.61	110	10.0	86.6	33.2
					25.0	94.6	29.9
					50.0	118	30.1
					100	106	9.78
					250	100	18.1
HT-2	0.74	1.48	38.4	89.2	2.50	44.7	55.9
					6.25	41.3	27.4
					12.5	49.2	21.7
					25.0	50.6	19.6
					62.5	42.5	19.7
NIV	0.68	1.36	124	98.7	10.0	86.1	38.6
					25.0	90.0	17.0
					50.0	99.2	12.9
					100	106	14.6
					250	93.6	12.3
OTA	0.18	0.36	27.5	107	0.50	90.9	24.5
					1.25	85.9	15.9
					2.50	91.3	13.9
					5.00	89.9	18.4
					12.5	84.5	6.97
OTα	0.14	0.28	19.4	87.7	0.50	124	30.9
					1.25	135	45.6
					2.50	86.9	35.3
					5.00	95.9	31.6
					12.5	79.5	28.7
ROQ-C	1.57	3.14	9.63	76.7	10.0	46.1	21.8
					25.0	59.6	19.7
					50.0	61.1	28.9
					100	58.8	24.1
					250	55.7	26.0
T-2	0.58	1.16	65.3	109	2.50	111	31.0
					6.25	107	13.7
					12.5	114	11.7
					25.0	109	9.13
					62.5	98.6	21.9
T-2 triol	1.26	2.52	47.2	92.1	10.0	80.4	46.4
					25.0	97.0	13.8
					50.0	122	15.2
					100	129	11.1
					250	116	14.9
ZEN	2.15	4.28	15.9	105	10.0	118	10.6
					25.0	98.4	19.3
					50.0	108	7.70
					100	106	15.2
					250	104	14.9

Aflatoxin B1 (AFB1), aflatoxin B2 (AFB2), aflatoxin G1 (AFG1), aflatoxin G2 (AFG2), aflatoxin M1 (AFM1), α-zearlenone (α-ZEL), alternariol (AOH), alternariol monomethyl ether (AME), β-zearalenone (β-ZEL), deepoxydeoxynivalenol (DOM-1), deoxynivalenol (DON), deoxynivalenol-3-glucoside (DON-3-glucoside), diacetoxyscirpenol (DAS), HT-2 toxin (HT-2), fumonisin B1 (FB1), fumonisin B2 (FB2), fumonisin B3 (FB3), nivalenol (NIV), ochratoxin A (OTA), ochratoxin α (OTα), roquefortin C (ROQ-C), T-2 toxin (T-2), HT-2 toxin (HT-2), T-2 triol toxin and zearalenone (ZEN).

**Table 3:** Apparent recovery (Rapp) for low, medium and high concentrations after 7 and 21 days at refrigeration (4 °C) and room temperature (20‒23 °C) for the analyzed mycotoxins: aflatoxin B1, aflatoxin B2, aflatoxin G1, aflatoxin G2, aflatoxin M1, alpha zearalenone, alternariol, alternariol methyl ether, beta zearalenone, deepoxy-deoxynivalenol, deoxynivalenol, deoxynivalenol-3-glucoside, diacetoxyscirpenol, fumonisin B1, fumonisin B2, fumonisin B3, HT-2 toxin, nivalenol, ochratoxin A, ochratoxin alpha, roquefortin C, T-2 toxin, T-2 triol toxin and zearalenone.

Mycotoxin	Spiked Concentration (ng/mL)	7 Days		21 Days	
		Room Temperature	4 °C	Room Temperature	4 °C
AFB1	0.5	129 ± 20	101 ± 34	116 ± 2	120 ± 15
	2.5	93.7 ± 8.7	98.8 ± 11.9	119 ± 5	121 ± 9
	12.5	99.5 ± 4.2	88.3 ± 10.5	86.3 ± 6.2	91.8 ± 4.9
AFB2	0.5	99.8 ± 20.2	138 ± 34	94.6 ± 15.6	118 ± 10
	2.5	105 ± 1	98.7 ± 9.9	106 ± 7	119 ± 16
	12.5	93.5 ± 10.7	101 ± 2	101 ± 7	91.0 ± 10.9
AFG1	0.5	32 ± 29 *	51.6 ± 15.3 *	107 ± 37	100 ± 1
	2.5	86.4 ± 1.4	91.8 ± 5.4	39 ± 25 *	112 ± 19
	12.5	99.0 ± 2.5	101 ± 5	72 ± 21	92.3 ± 6.2
AFG2	0.5	106 ± 7	108 ± 7	85.9 ± 15.2	82.7 ± 11.4
	2.5	92.1 ± 2.4	87.4 ± 2.0	105 ± 5	91.8 ± 10.3
	12.5	85.8 ± 2.0	88.8 ± 2.9	92.3 ± 10.9	112 ± 21
AFM1	0.5	109 ± 15	143 ± 17	110 ± 2	115 ± 15
	2.5	103 ± 15	122 ± 2	104 ± 1	90.4 ± 10.9
	12.5	105 ± 4	114 ± 3	104 ± 4	90.9 ± 11.9
α-ZEL	10	106 ± 36	120 ± 19	82.9 ± 17.4	101 ± 16
	50	81.2 ± 9.9	90.5 ± 11.0	117 ± 14	94.5 ± 1.3
	250	121 ± 16	98.3 ± 11.8	120 ± 10	80.3 ± 2.4
AOH	10	118 ± 17	94.2 ± 1.7	92.6 ± 10.3	91.0 ± 12.3
	50	90.5 ± 2.9	83.3 ± 12.8	98.1 ± 18.7	90.5 ± 11.9
	250	78.7 ± 1.8	76.1 ± 3.3	96.8 ± 12.3	80.9 ± 12.8
AME	10	89.3 ± 10.5	98.5 ± 27.8	83.6 ± 12.7	85.1 ± 13.4
	50	83.6 ± 1.5	82.6 ± 2.1	82.6 ± 13.5	89.5 ± 11.3
	250	76.5 ± 3.8	77.8 ± 2.1	81.8 ± 4.9	98.7 ± 4.2
β-ZEL	10	138 ± 22	108 ± 19	93 ± 22	121 ± 15
	5 0	129 ± 9	99.5 ± 11.3	145 ± 18	110 ± 11
	250	122 ± 10	115 ± 12	142 ± 20	119 ± 24
DOM-1	2.5	106 ± 16	110 ± 10	101 ± 11	119 ± 9
	12.5	108 ± 1	94.0 ± 10.9	85.6 ± 20.6	89.5 ± 20.1
	62.5	115 ± 11	114 ± 12	108 ± 4	81.3 ± 11.9
DON	2.5	94.9 ± 13.6	120 ± 22	98.3 ± 35.7	109 ± 19
	12.5	88.4 ± 13.3	106 ± 2	90.2 ± 3.5	99.5 ± 11.3
	62.5	99.9 ± 10.9	98.7 ± 7.4	94.8 ± 4.9	98.3 ± 2.4
DON-3-glucoside	2.5	75.5 ± 15.2	87.6 ± 9.1	75.6 ± 25.2	81.8 ± 9.1
	12.5	80.2 ± 10.5	91.5 ± 10.1	119 ± 30	90.9 ± 19.3
	62.5	85.7 ± 14.8	100 ± 16	128 ± 28	91.6 ± 15.1
DAS	2.5	110 ± 16	90.2 ± 1.4	81.6 ± 10.7	84.1 ± 15.3
	12.5	106 ± 12	110 ± 9	92.2 ± 13.5	90.6 ± 19.1
	62.5	109 ± 94	115 ± 12	104 ± 11	85.7 ± 0.2
FB1	10	77.5 ± 16.9	77.1 ± 16.5	81.3 ± 4.2	67.5 ± 6.1
	50	99.5 ± 17.2	91.6 ± 10.6	89.8 ± 7.6	95.5 ± 4.6
	250	106 ± 21	103 ± 23	92.2 ± 2.7	93.0 ± 8.9
FB2	10	101 ± 16	115 ± 11	42.0 ± 20.9	48.1 ± 20.6
	50	79.2 ± 13.0	112 ± 6	97.6 ± 30.6	91.8 ± 10.1
	250	80.8 ± 18.2	80.1 ± 9.6	77.6 ± 20.8	78.3 ± 12.2
FB3	10	113 ± 10	110 ± 19	101 ± 26	101 ± 9
	50	120 ± 19	119 ± 20	55.6 ± 23.5 *	69.5 ± 10.9
	250	120 ± 18	90.5 ± 15.4	53.2 ± 34.9 *	70.1 ± 12.4
HT-2	2.5	35.7 ± 40.5	45.3 ± 5.7	45.6 ± 3.9	46.6 ± 9.9
	12.5	42.8 ± 7.2	49.2 ± 9.5	39.6 ± 4.5	49.5 ± 1.3
	62.5	42.9 ± 5.5	50.2 ± 12.1	37.1 ± 4.3	48.3 ± 20.4
NIV	10	80.2 ± 12.2	119 ± 14	97.6 ± 18.6	118 ± 5
	50	81.1 ± 19.5	110 ± 20	80.6 ± 12.6	81.9 ± 1.0
	250	95.6 ± 16.2	103 ± 12	109 ± 16	100 ± 4
OTA	0.5	117 ± 15	89.2 ± 14.2	106 ± 4	119 ± 1
	2.5	109 ± 8	99.9 ± 6.6	109 ± 3	91.5 ± 11
	12.5	90.5 ± 3.1	88.1 ± 1.9	103 ± 2	90.1 ± 12.8
OTα	0.5	128 ± 36	97.5 ± 4.6	116 ± 18	102 ± 14
	2.5	89.2 ± 3.5	92.0 ± 8.6	82.6 ± 12.4	106 ± 19
	12.5	101 ± 5	91.6 ± 9.1	118 ± 11	93.2 ± 12.0
ROQ-C	10	70.2 ± 22.9	70.6 ± 19.4	84.6 ± 19.5	81.1 ± 10.9
	50	41.2 ± 20.2	95.5 ± 14.8	87.9 ± 10.6	81.5 ± 6.5
	250	103 ± 16	75.6 ± 16.2	111 ± 15	89.1 ± 4.1
T-2	2.5	40.0 ± 23.5 *	45.8 ± 18.2 *	90.5 ± 14.2	78.6 ± 15.1
	12.5	89.9 ± 6.3	74.1 ± 7.0 *	111 ± 7	82.6 ± 11.3
	62.5	85.8 ± 9.3	79.3 ± 6.5	116 ± 17	81.3 ± 9.9
T-2 triol	10	81.7 ± 6.7	88.4 ± 11.5	82.6 ± 7.2	118 ± 12
	50	104 ± 7	120 ± 7	107 ± 15	91.1 ± 8.2
	250	102 ± 8	111 ± 8.2	96.9 ± 10.4	97.9 ± 4.0
ZEN	10	98.8 ± 3.6	98.8 ± 1.1	98.1 ± 1.2	116 ± 22
	50	103 ± 2	101 ± 1	103. ± 3.5	80.4 ± 20.8
	250	104 ± 5	100 ± 5	101 ± 3	90.1 ± 17.9

Aflatoxin B1 (AFB1), aflatoxin B2 (AFB2), aflatoxin G1 (AFG1), aflatoxin G2 (AFG2), aflatoxin M1 (AFM1), α-zearlenone (α-ZEL), alternariol (AOH), alternariol monomethyl ether (AME), β-zearalenone (β-ZEL), deepoxydeoxynivalenol (DOM-1), deoxynivalenol (DON), deoxynivalenol-3-glucoside (DON-3-glucoside), diacetoxyscirpenol (DAS), HT-2 toxin (HT-2), fumonisin B1 (FB1), fumonisin B2 (FB2), fumonisin B3 (FB3), nivalenol (NIV), ochratoxin A (OTA), ochratoxin α (OTα), roquefortin C (ROQ-C), T-2 toxin (T-2), HT-2 toxin (HT-2), T-2 triol toxin and zearalenone (ZEN). *: reduction in the recovery compared to “fresh samples” with a statistical difference as indicated by *p* < 0.05.

**Table 4:** Mycotoxin presence (%) >LOD, average and standard deviation (ng/mL) and maximum concentration (ng/mL) in 20 blood samples analysed using the VAMS and liquid/liquid extraction method.

Mycotoxin	VAMS			Liquid/Liquid		
	Presence (%)	Average ± SD (ng/mL)	Max. (ng/mL)	Presence (%)	Average ± SD (ng/mL)	Max. (ng/mL)
AFB1	10	0.10 ± 0.06	0.16	10	0.09 ± 0.08	0.12
α-ZEL	0	n.a.	n.a.	5	2.71	n.a.
OTA	60	0.56 ± 0.12	0.71	75	0.42 ± 0.18	0.76
OTα	80	0.83 ± 0.21	1.14	80	0.78 ± 0.29	1.28
ZEN	10	8.05 ± 5.02	14.02	10	7.68 ± 4.81	13.26

Ochratoxin A (OTA), ochratoxin alpha (OTα), zearalenone (ZEN), α-zearlenone (α-ZEL) and aflatoxin (AFB1). n.a. = not applicable.
